# Considering SOD and miRNA analysis as potential prognostic markers in white lesion malignant transformation: A report of two cases

**DOI:** 10.1097/MD.0000000000040928

**Published:** 2024-12-13

**Authors:** Zuzana Drotárová, Miroslava Rabajdová, Mária Mareková, Peter Kizek, Janka Vašková

**Affiliations:** a Department of Stomatology and Maxilofacial Surgery, L. Pasteur University Hospital, Košice, Slovak Republic; b Department of Medical and Clinical Biochemistry, Faculty of Medicine, Pavol Jozef Šafárik University in Košice, Košice, Slovak Republic; c Department of Medical Biology, Faculty of Medicine, Pavol Jozef Šafárik University in Košice, Košice, Slovak Republic.

**Keywords:** antioxidant enzyme, biomarker, malignant transformation, miRNA, oral leukoplakia, reduced glutathione

## Abstract

**Rationale:**

Predictive biomarkers can be effective in the identification of the oral leukoplakia with an increased probability of malignant transformation.

**Patients concerns:**

A 63-year-old patient presents with persistent burning sensations throughout the oral cavity, accompanied by a white lesion on the tongue. Additionally, a 57-year-old patient with multiple white lesions in the oral cavity.

**Diagnosis:**

Histopathological analysis of the excised tissue.

**Interventions:**

Changes in the expression miRNAs (miR17, miR206, and miR23), the activity of antioxidant enzymes (superoxide dismutase, glutathione peroxidase, glutathione reductase, and glutathione-S-transferase), and concentration of reduced glutathione were detected, followed by meta-analysis of previous studies.

**Outcomes:**

In both patients (verrucous leukoplakia, oral squamous cell carcinoma) upregulated expression of miR-23a, miR-17, and downregulated expression of miR206 were detected when compared to healthy individuals. In the plasma of a patient diagnosed with carcinoma, higher activity of antioxidant enzymes connected to glutathione was measured in comparison to healthy individuals.

**Lessons:**

The connection between miRNA expression changes, the increase in glutathione-S-transferase and especially the decrease in superoxide dismutase activities in patients with white lesion potential malignant transformation using the provided statistical analysis was confirmed.

## 1. Introduction

Oral squamous cell carcinoma (OSCC) is the most common head and neck malignancy with a global incidence of >300,000 new cases per year, making it the sixth leading cause of morbidity and mortality.^[1]^ OSCC also accounts for up to 90% of all neoplasias affecting the oral mucosa.^[2]^ Carcinoma often develops from preexisting, potentially premalignant disorders such as oral leukoplakia, submucosal fibrosis, erythroplakia and accounts for 30% to 40% of all cancers.^[3,4]^ Smoking, chewing tobacco, alcohol, poor hygiene in the hospital, but also genetic predisposition and malnutrition are among the main etiological factors of OSCC.^[5]^ Oral leukoplakia is the most common premalignant lesion of the oral mucosa and is defined as “a white plaque that cannot be clinically or pathologically characterized as any other disease.”^[6]^ Study of Ganjre and Bagul demonstrated that up to 16% to 62% of oral carcinomas are associated with leukoplakia lesions, and we can argue that precancerous lesions can act as a prognostic, clinical marker for oral carcinoma screening.^[6]^

Several studies also describe the association between oxidative stress and the precancerous lesions themselves, or the development of the OSCC.^[3,4,7]^ Oxidative stress is also implicated in the development and progression of various other oral pathologies, such as lichen planus, recurrent aphthous lesions, or the occurrence of periodontal disease.^[8,9]^ Antioxidant enzymes prevent carcinogenesis in early stages, however, in later stages, high activity is undesirable, as they enable tumor progression by lowering oxidative stress conditions.^[6]^ Cancer cells have a high cholesterol concentration and a high cholesterol/phospholipid ratio, which means that damage to membrane lipids by peroxidation is considerably limited in tumor cells.^[10]^ Increased levels of lipid peroxidation products and reduced content of antioxidant enzymes in the blood can be observed in patients with precancerous lesions, but also in healthy individuals who regularly chew tobacco and smoke, which may be useful in predicting the risk of the oral cancer.^[11]^

Important regulatory elements that intervene in almost every physiological function of the cell, are miRNAs.^[12]^ Study of Cervigne provided a miRNA signature as potentially useful for identifying leukoplakias at risk of malignant transformation.^[13]^ Also, other studies showed the differential expression of miRNAs in the plasma of patients with OSCC compared to healthy subjects.^[14–17]^ The changes in miRNAs in the blood related to some disease may not be identical to the changes in miRNAs in cancer tissues.^[18]^ Several studies analyze the influence of different groups of miRNAs on precancerous and carcinogenic lesions.^[13,19–21]^ Cancer cells can selectively release specific miRNAs via exosomes into the extracellular environment, therefore miRNA expression patterns in a tumor sample and serum from the same patient may not correlate. In a meta-analysis of 13 independent miRNA profiling studies of head and neck squamous cell carcinoma, 432 miRNAs were found to be differentially expressed, 90 of which were reported in at least 2 studies.^[21]^ Individual miRNA profiles correlate with disease etiology, pathogenesis, metastasis and recurrence of head and neck squamous cell carcinoma.

Clinical and histological characteristics have limited prognostic value, making it difficult to predict which leukoplakias will progress to a tumor. We identified 3 miRNAs that have been shown in several of the aforementioned studies to significantly impact precancers. Additionally, we selected antioxidant markers from 2 patients to facilitate analysis and comparison with prior research, aiming to identify parameters with prognostic significance.

## 2. Materials and methods

### 2.1. Sampling

**Figure 1.**
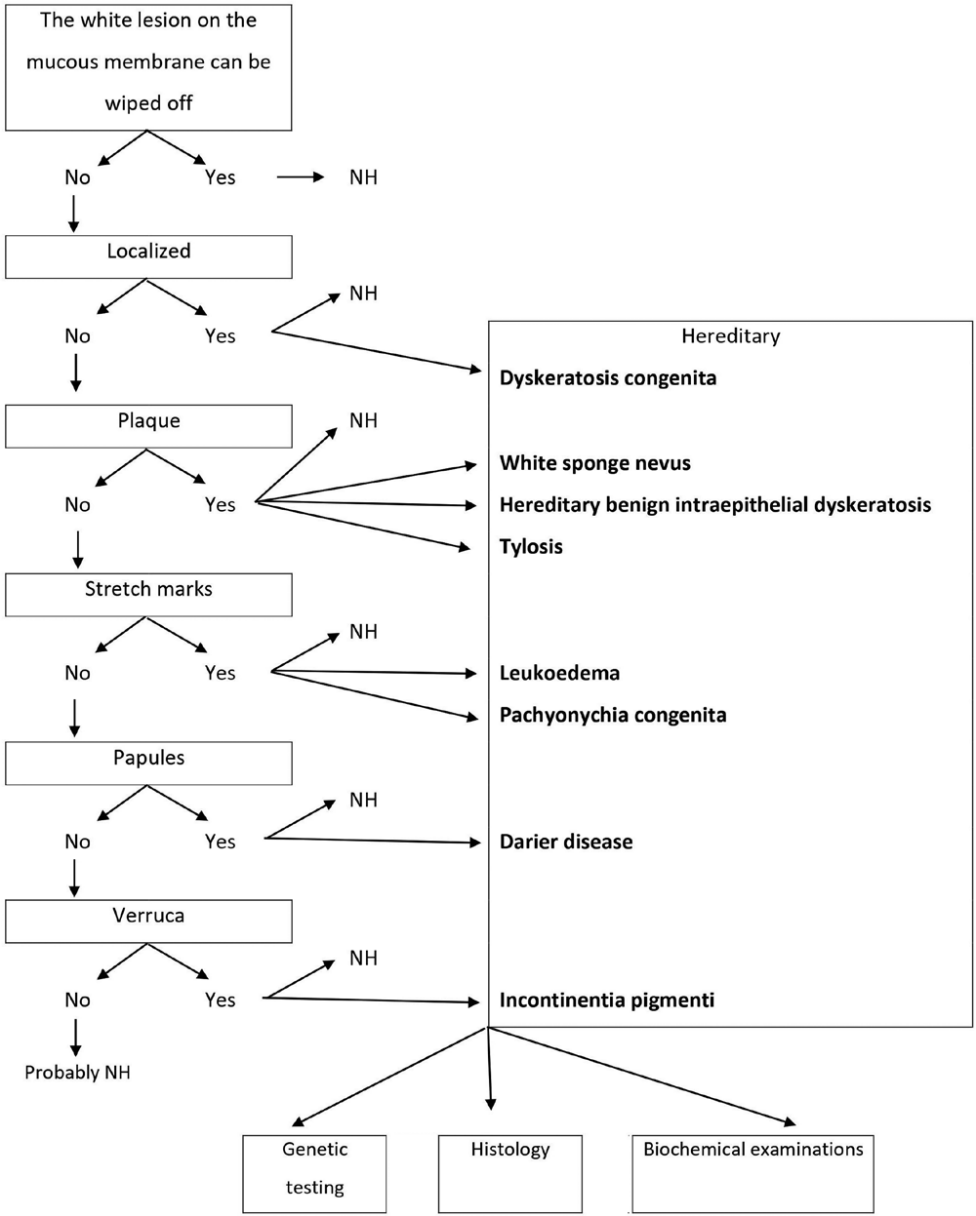
Diagnostic model of white lesions of the oral mucosa. NH = nonhereditary.

The study received approval from the Ethics Committee of L. Pasteur University Hospital under number 17/EK/19. Signed informed consent was obtained from both patients and probands. Patient selection utilized a diagnostic model, enhanced by biochemical examination (see Fig. 1). The study included 2 patients for whom the model did not exclude the possibility of a hereditary white lesion in the oral cavity, as indicated by their medical history and clinical examination.

### 2.2. miRNA isolation and analysis

MiRNA was isolated from plasma samples using a column-based commercial AllPrep DNA/RNA/miRNA Universal Kit (Qiagen) following the manufacturer’s instructions. The concentration of isolated RNA was measured at wavelengths λ = 260 and 280 nm on a Nanodrop 2000C spectrophotometer. RNase-free water was used as a blank. Optimal purities for the 260/280 nm ratio are in the range of 1.8 to 2.0. Samples were diluted to a concentration of 6.67 ng/3.33 µL. RNA samples with a given concentration were used for transcription into cDNA. The remaining volume of samples was stored at −70°C. Subsequently, the miRNA was transcribed into cDNA using a TaqMan® MicroRNA Reverse Transcription Kit (Applied Biosystems, Waltham, MA). Quantitative PCR (qPCR) was performed using a TaqMan® Universal MM, no UNG (Applied Biosystems, Waltham, MA) on a Rotorgene (Qiagene, Hilden, Germany).

### 2.3. Antioxidant markers analysis

Superoxide dismutase (SOD) determination kit, Glutathione Peroxidase (GPx) Cellular Activity Assay kit, Glutathione Reductase (GR) Assay Kit and Glutathione-S-transferase (GST) Assay Kit (all Sigma Aldrich, Taufkirchen, Germany) were used for the detection of SOD, GPx, GR, and GST by kinetic methods. Reduced glutathione (GSH) content was measured by the method originally described by Floreani et al.^[22]^ Assays were performed on an M 501 single beam UV/VIS spectrophotometer (Spectronic Camspec Ltd., Leeds, United Kingdom). All measured parameters were calculated per milligram of protein determined using the bicinchoninic acid assay.

### 2.4. Statistical analysis

The quantitative analysis method according to Whitehead was used.^[23]^ For the analysis, we selected a total of 8 studies (S_1_–S_8_), partially addressing the issue of the relationship between proven OSCC and the level of selected antioxidants in the blood. We denoted our own results with S_0_ (Table 1).

**Table 1:** Designation of analyzed studies.

Study	Source
*S*_*0*_	Cases in this study
*S*_*1*_	^[2]^
*S*_*2*_	^[24]^
*S*_*3*_	^[25]^
*S*_*4*_	^[26]^
*S*_*5*_	^[27]^
*S*_*6*_	^[5]^
*S*_*7*_	^[28]^
*S*_*8*_	^[29]^

S = study.

### 2.5. Case presentations

#### 2.5.1. Patient A

**Figure 2.**
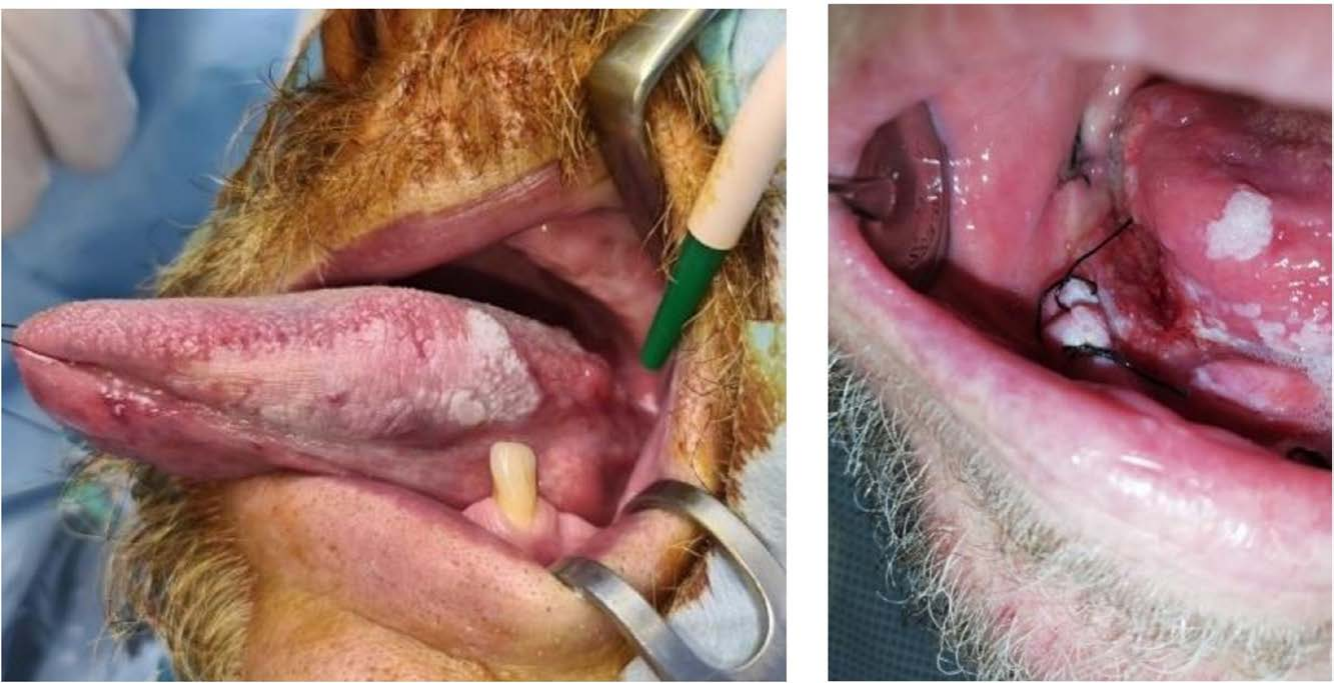
Left: A white, sharply defined lesion on the margo linguae l.sin. in patient A. Right: Multiple white lesions and a suspected ulcerative lesion on the floor of the oral cavity in patient B.

A 63-year-old patient was referred to the periodontology department due to persistent burning sensations throughout the oral cavity, particularly on the tongue. At the time of assessment, the patient reported no other subjective complaints or known allergies. He acknowledged a history of nicotine use and occasional alcohol consumption, but denied the use of other addictive substances. The patient was being treated for hypertension and was prescribed Amiesa. He reported the presence of a white patch on the left edge of his tongue, which had persisted for 3 years and was being monitored by his dentist. No similar lesions or abnormalities were noted on the nasal, laryngeal, or anal mucosa. Family history could not be confirmed. The patient presented with an edentulous jaw and a single, rare tooth remaining in its socket. The mucous membranes appeared pink, moist, and physiologically coated, without any pathological lesions or other abnormalities. The tongue exhibited a prominent white coating, was drier, slightly furrowed, and had a midline fissure, but retained intact mobility. The white coating on the root of the tongue could be wiped off; however, along the lateral margin of the tongue, there was a non-wipeable, painless, non-bleeding lesion that was elevated above the surrounding mucosa (Fig. 2, left). The dorsal aspect of the lesion appeared verrucous in nature, and a culture and sensitivity smear confirmed the presence of an infection. The patient was treated with Fluconazole for 14 days. 2 weeks later, a follow-up swab was taken from the tongue, which indicated that the infection had cleared. The tongue had a slight white coating, but the white lesion on the left lateral margin persisted. This lesion measured approximately 7 × 2 cm, had sharply defined edges, and exhibited a verrucous appearance at the base near the root of the tongue (Fig. 2, left). The patient subsequently underwent excision of the entire white area with a safety margin. A portion of the left lateral margin of the tongue was preserved in 10% formalin. The resection size was approximately 25 × 22 × 8 mm, with a vaguely defined white area measuring 15 × 11 mm on the surface. The sample was sent for histological examination, and the tongue was sutured in 2 layers. The patient was prescribed Zinnat 500 mg tablets for 7 days. Sutures were removed on the tenth day post-surgery, and the wound was treated locally with 1.5% hydrogen peroxide. Histopathological analysis confirmed the diagnosis of verrucous leukoplakia, with no evidence of lichenoid inflammatory changes or mycosis. A follow-up examination was conducted 2 months after the excision of the lesion on the left lateral margin of the tongue. During this visit, the wound appeared calm, with no signs of inflammation, no pain upon palpation, and no other pathological findings.

#### 2.5.2. Patient B

During the intraoral examination of a 57-year-old patient, multiple white lesions (Fig. 2, right) were identified. The patient had a long-standing history of treatment for hypothyroidism and hypertension, with no reported allergies. He has been smoking 20 cigarettes a day since the age of 18 and consumes alcohol occasionally. Several teeth had been extracted, and a suture was present in the oral cavity at the time of examination. The examination revealed multiple white, non-wipeable lesions extending across the base of the tongue. These lesions exhibited a verrucous appearance, were sharply demarcated, painless, and did not bleed upon stimulation. Mild signs of inflammation were observed at the base of the tongue, where an ulcerous lesion was also present. The mobility of the tongue remained unaffected, and it exhibited a central midline fissure. A biopsy of the white lesions indicated clinical signs suggestive of a potential cancerous process. Histological examination confirmed infiltration by squamous cell carcinoma structures. The patient was subsequently diagnosed with OSCC at the base of the tongue, classified as stage T3, and was recommended for radiotherapy.

## 3. Results

The evaluated antioxidants in the plasma were compared with the values of a control group consisting of 12 healthy individuals (Table 2). In patient B, a higher activity of GPx, GR, GST, but also a significantly higher level of GSH compared to the control group was detected. Changes in the expression of the 3 mentioned miRNAs (miR17, miR206, and miR23) in the patients’ blood were found and compared with a control group without a previous positive family history and with a negative preventive examination by a doctor (n = 3). As shown in Table 3, miR-23a and miR-17 were upregulated and miR206, was downregulated.

**Table 2:** Measured antioxidants parameters in the plasma of patients.

	SOD (U/mg prot)	Se-GPx (U/mg prot)	GR (U/mg prot)	GST (U/mg prot)	GSH (U/mg prot)
Control	4.62 ± 1.98	17.73 ± 1.60	12.73 ± 1.45	1.53 ± 0.21	3.39 ± 0.35
Patient A	5.88	18.33	8.28	1.04	3.46
Patient B	3.52	24.32	23.56	2.47	21.39

GR = glutathione reductase, GSH = reduced glutathione, GST = glutathione-S-transferase, Se-GPx = selenium containing glutathione peroxidase, SOD = superoxide dismutase.

**Table 3:** Changes in the expression of miR17, miR206, and miR23a in the plasma of our patients.

		Ct	ΔCt exp	ΔΔCt exp	2−^ΔΔCt^ (RQ)	Result
miR206	Patient A	37.5	−2.50	1.30	0.41	Downregulated
	Patient B	37.4	−2.60	1.20	0.44	Downregulated
	Control	36.2	−3.80			
miR23a	Patient A	30.4	−9.60	−3.80	13.93	Upregulated
	Patient B	29.2	−10.80	−5.00	32.00	Upregulated
	Control	34.2	−5.80			
miR17	Patient A	25.5	−14.50	−2.60	6.06	Upregulated
	Patient B	25.3	−14.70	−2.80	6.96	Upregulated
	Control	28.1	−11.90			

**Figure 3.**
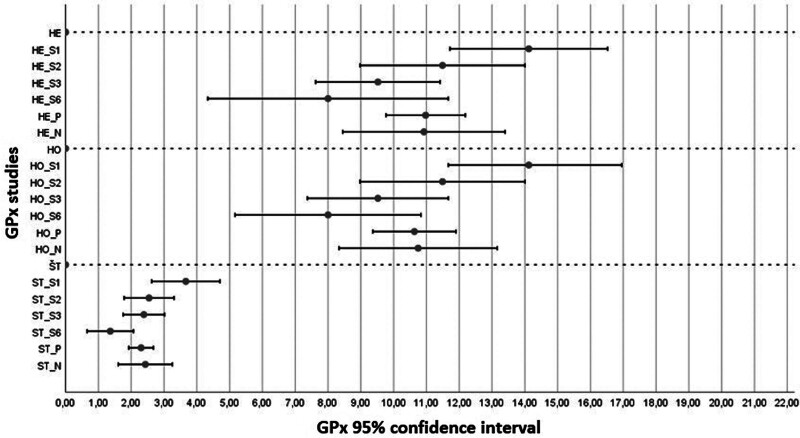
Meta-analysis results for the antioxidant glutathione peroxidase (GPx). The x-axis is common to all parts. The abscissa represents the 95% confidence interval (highlighted value estimate of the mean value $\hat{\theta }$) of the difference between the research and control samples. The labels S1 to S8 present individual studies relevant to the analyzed antioxidant. These are values: estimation of fixed effects assuming heterogeneity of studies (HE), homogeneity of studies (HO) and standardization (ST). Labeled P: for the fixed effects model and N: for the random effects model, the relevant pooled 95% confidence intervals are presented, and the highlighted value is the estimate of the overall mean $\hat{\theta }$. Statistical tests performed: *U* test for significance of differences between research and control samples and Q-test of homogeneity between studies for differences between control and research samples.

A comparison of the antioxidants activities in the studies between patients with OSCC and healthy subjects was performed (Table 4). All tests of the U statistic for GPx, as well as the results of our analysis (Fig. 3), clearly confirm a statistically significant increase in GPx in OSCC patients. The Q statistic test confirmed heterogeneity between studies, thus we used a random model for the evaluation of the effect. The estimate of the mean value of the increase in the research sample from the control represented an average value of 10.75 under the assumption of homoscedasticity and 10.92 under the assumption of heteroskedasticity. The 95% confidence intervals were very similar. The biggest differences were between studies S_1_ and S_6_. The standardized HO estimate indicated that at 95% confidence probability we could expect a significant shift in the mean value of GPx activity in OSCC patients at the level of 1.6 to 3.25 times the standard deviation of measurement.

**Table 4:** Comparison of the results of superoxide dismutase (SOD), glutathione peroxidase (GPx), glutathione reductase (GR), glutathione-S-transferase (GST) enzyme activities and the level of reduced glutathione (GSH) of the patients A, B with patients with oral cancer in other studies and control group. Studies marked with an asterisk are those whose measured data most closely match the results of our measurements (study S0). C: control, M: patients with malignity.

	Study	Patient A	Patient B	Control	OSCS patients	*P*-value	Patients
SOD	S_0_	5.88	3.52	4.62 ± 1.98			C-12, M-2
	S_4_*			4.45 ± 2.31	2.45 ± 1.21	.053	C-25, M-18
	S_1_			19.37 ± 3.09	8.55 ± 1.20	<.05	C-20, M-18
	S_2_			16.65 ± 3.36	13.18 ± 3.97	<.001	C-24, M-24
	S_5_			4.37 ± 1.43	1.49 ± 0.49	<.005	C-102, M-100
GPx	S_0_	18.33	24.32	17.73 ± 1.60			C-12, M-2
	S_2_*			17.11 ± 3.18	28.6 ± 5.42	<.001	C-24, M-24
	S_1_			12.79 ± 2.14	26.91 ± 5.00	<.001	C-20, M-18
	S_6_			7.96 ± 2.09	15.96 ± 7.67	<.001	C-18, M-20
	S_3_			13.27 ± 3.83	22.79 ± 4.03	<.001	C-33, M-33
GR	S_0_	8.28	23.56	12.73 ± 1.45			C-12, M-2
	S_1_			11.21 ± 1.44	23.22 ± 4.24	<.001	C-20, M-18
	S_7_			31.46 ± 18.05	74.5 ± 41.5	<.001	C-50, M-50
GST	S_0_	1.03	2.47	1.53 ± 0.21			C-12, M-2
	S_3_*			1.24 ± 0.21	2.25 ± 0.28	<.001	C-33, M-33
	S_8_*			1.39 ± 0.54	3.43 ± 1.93	.05	C-10, M-14
GSH	S_0_	3.46	21.39	3.39 ± 0.35			C-12, M-2
	S_1_*			4.39 ± 0.90	25.71 ± 4.98	<.001	C-20, M-18
	S_2_			6.32 ± 1.82	17.4 ± 4.27	<.001	C-24, M-24

GPx = selenium containing glutathione peroxidase, GR = glutathione reductase, GSH = reduced glutathione, GST = glutathione-S-transferase, SOD = superoxide dismutase.

The results of the analysis of GR were based on studies S_1_ and S_7_. The data did not indicate significantly higher values of GR in OSCC patients compared to the control group in both studies. The Q-test indicated significant heterogeneity between studies, thus we preferred random effects estimation. The hypothesis of a significant difference between the research and control samples at the 5% level of significance (except for the case of homogeneity of studies standardization, where the *P*-value was .042) was rejected. The 95% confidence interval was for N random-effects disproportionately large relative to the others, confirming the ambiguity of analysis interpretation.

The *U* test for GSH proved the significance of the differences between the research and control samples except the cases of standardization of estimates. The Q-test indicated significant heterogeneity between studies, also reflected in the difference in the 95% confidence intervals for P fixed and N random effects. An inhomogeneity between studies was significantly reflected in the standardized estimates, both for fixed and random effects. *U* tests for standardized HO estimates indicated non-significance of differences between research and control samples for both types of effects reflecting a certain disproportionality of measurements between individual studies.

**Figure 4.**
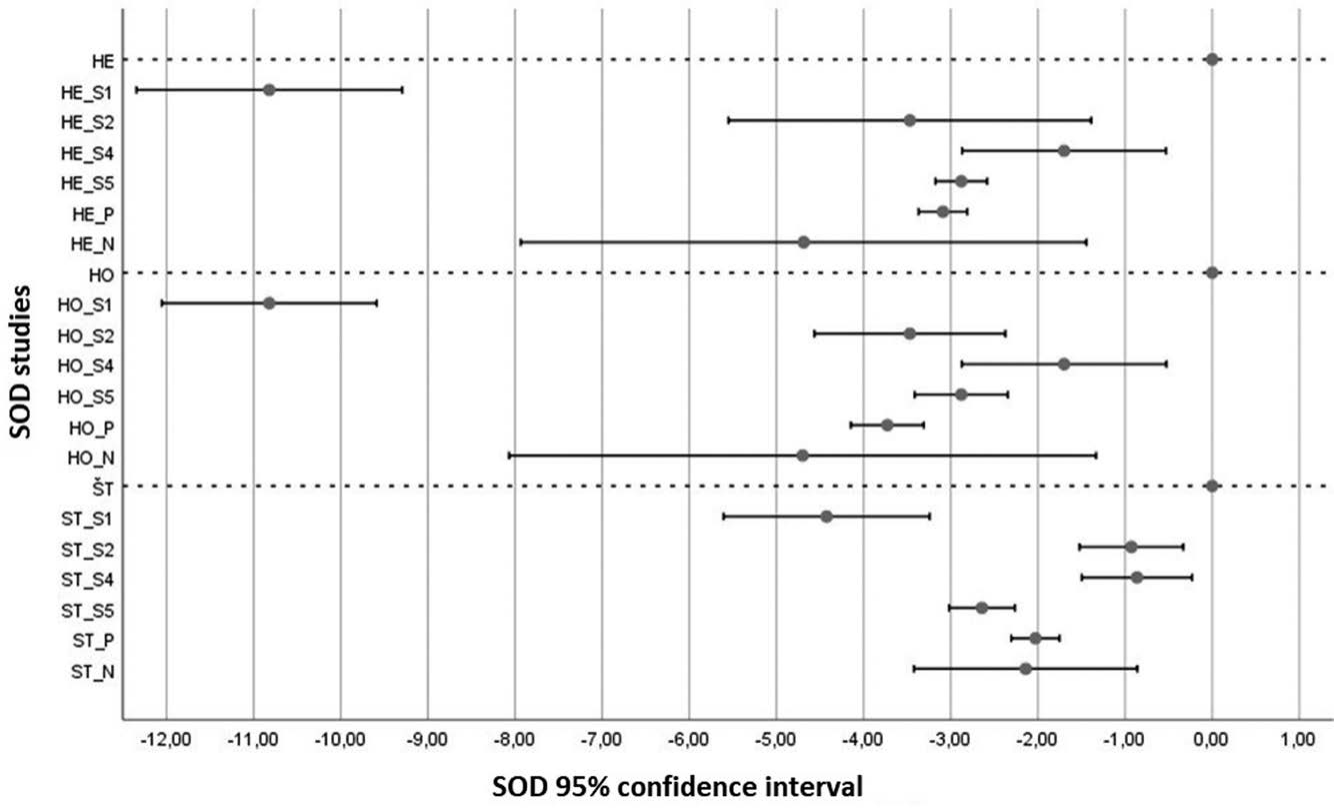
Meta-analysis results for superoxide dismutase (SOD). The x-axis is common to all parts. The abscissa represents the 95% confidence interval (highlighted value estimate of the mean value $\hat{\theta }$) of the difference between the research and control samples. The labels S1 to S8 present individual studies relevant to the analyzed antioxidant. These are values: estimation of fixed effects assuming heterogeneity of studies (HE), homogeneity of studies (HO) and standardization (ST). Labeled P: for the fixed effects model and N: for the random effects model, the relevant pooled 95% confidence intervals are presented, and the highlighted value is the estimate of the overall mean $\hat{\theta }$. Statistical tests performed: *U* test for significance of differences between research and control samples and Q-test of homogeneity between studies for differences between control and research samples.

The results of all *U* tests for SOD clearly declared a significant decrease of SOD in the blood of OSCC patients against the control group (Fig. 4). The 4 analyzed studies showed significant difference between-group heterogeneity by the Q-test, expressed by the difference in the sizes of the 95% confidence intervals for P fixed and N random effects. A significant decrease in SOD between the research and control samples by an average of 1.33 to 8.06 could be noted in the estimations of all models.

A Q-test for GST on fixed effects without the assumption of common variance at the 5% significance level rejected the hypothesis of study heterogeneity. Relevant *U* tests confirmed significantly higher GST values in the blood of OSCC patients compared to the control group. An average increase of GST in the blood at a level from about 0.4 to 2.3 could be expected. Standardized HO estimate for random effects performed (*P*-value of the *U* test was .05098) was due to the enormous difference in the variance of the measurements between the studies.

## 4. Discussion

The aim of this study was to evaluate the potential of measuring antioxidant levels and specific miRNAs for early prediction of malignant transformation in white lesions of the oral cavity. These molecules can regulate over half of the coding genes in the human genome, with each miRNA capable of targeting hundreds of mRNAs.^[30]^ While deregulation of miRNAs does not affect the growth of normal cells, it restricts the proliferation of cancer cells, indicating that miRNAs could serve as therapeutic targets for cancer treatment.^[31]^ MiRNAs are classified as either tumor suppressor or oncogenic miRNAs based on their expression and function in cancer tissues. Given the varying expression profiles of miRNAs across different cancer types, they can also be considered specific biomarkers for certain cancers.^[32]^ The identification of specific serum miRNAs associated with precancerous lesions further supports their potential role in the early detection of oral cancer. Due to the relatively small ranges, it was very difficult to estimate the degree of homogeneity of the measured data in all studies. In accordance with the methodology presented by Whitehead, after the logarithmic transformation, the data were treated as normally distributed.^[23]^ The results of our analysis were consistent with those published for oral cancer patients, showing that miR-23a and miR-17 were upregulated while miR-206 was downregulated.^[21]^ However, the miRNA analysis did not reveal significant differences between patients A and B, unlike the findings related to certain antioxidants. Due to the small sample size, the miRNAs we measured were unable to distinguish the potential transformation of the white lesion to carcinoma in either patient.

Changes in biochemical markers in patients’ serum during the early stages of disease can aid in early diagnosis, treatment selection, and disease prognosis.^[9,33]^ In patient B, we observed increased activities of GPx, GR, and GST, along with elevated GSH concentration (Table 2). Increased GPx activity in whole blood and tissue samples from patients with OSCC has also been reported by Deshpande et al, leading to its consideration as a prognostic marker for cancer progression across various stages.^[5]^ Moreover, the mean GR activity in the serum of patients with tumors was significantly higher compared to the control group.^[28]^ We also noted reduced SOD activity in patient B, a finding consistent with several other studies.^[24,34–37]^ Elevated antioxidant capacities in cancer tissues may render them less susceptible to oxidative stress, providing a selective growth advantage to tumor cells. These results suggest that the reduction in SOD activity, coupled with increases in GSH, GPx, and GR levels in oral tumor tissue, may represent an adaptive mechanism that allows tumor cells to gain a selective advantage over surrounding normal cells.^[2]^ Highly aggressive tumors frequently overexpress cytoprotective enzymes, including GST, contributing to their resistance to various chemotherapeutic agents. This was not the case for patient A, whose diagnosis reflects only a potential transformation state.

Since the analyses did not yield statistically significant results due to the small patient sample, we incorporated findings from existing studies into our evaluation (Table 4). In cases of rare diseases, it is common to utilize multicenter study approaches alongside meta-analyses.^[38,39]^ Research indicates that patients with oral cancer exhibit significantly reduced SOD activity compared to healthy individuals.^[27]^ Available data suggest that tumorigenesis is linked to decreased SOD levels.^[2,24,26,34–36]^ The increased activities of GPx observed in our study align with findings from other published research.^[2,10,24–26,35,37]^ An elevation in GPx levels in tumor tissue has been documented in studies examining patients with OSCC, as well as in colorectal, lung, breast, cervical, laryngeal, and hepatocellular cancers.^[5]^ Additionally, mean levels of GR in the serum and tumor tissue of patients were significantly higher compared to those in the control group.^[2,28]^ Glutathione serves as a cofactor for GPx and plays a regulatory role in cell proliferation; it is often overexpressed in malignant tumors. Studies have shown that GSH levels can be up to 5 times higher in tumor tissue compared to healthy tissue.^[2,24]^ The primary function of GST is to detoxify carcinogens. Notably, individuals with a null phenotype for certain GST isoforms have an increased risk of developing cancer. Elevated GST activity has been observed in OSCC tissues compared to normal mucosa.^[29]^ GST upregulation has been documented in both precancerous and malignant lesions of the oral cavity. Low GST levels may facilitate the initiation phase of carcinogenesis associated with mutations. In patient A, reduced GST activity was detected, aligning with his diagnosis of verrucous leukoplakia, a condition known to have a propensity for progression to oral carcinoma. Thus creating the conditions for the process of carcinogenesis, but not characterizing the tumor itself. Meanwhile, the increase in GST observed in OSCC-related oral lesions may enhance tumor cell survival during cancer progression. Highly progressive tumors often overexpress cytoprotective enzymes, including GST, which are partially responsible for their resistance to various chemotherapeutic agents. The premalignant and malignant oral lesions undergo biochemical adaptation, resulting in increased GST levels.^[29]^ An increase in GST was also described by Gurudath et al.^[40]^

Statistical comparisons of studies suggest that the reduction in SOD activity, alongside increases in GSH, GPx, and GR levels in oral tumor tissue, may represent an adaptive mechanism for tumor cells. However, for GSH, we cannot definitively confirm this due to significantly differing results from standardized assessments. Similarly, our analysis did not convincingly establish an increase in GR concentration in the blood of OSCC patients compared to the control group. For GST, despite substantial variability in measurements across studies, our analysis confirmed an increase in enzyme levels in the research sample relative to the control sample. The results for SOD and GST clearly align with previously published findings. In our miRNA analysis, we were unable to reliably determine whether there was a potential for the conversion of the white lesion into oral cavity cancer.

## 5. Conclusion

Our study expanded the diagnostic model we proposed by incorporating the analysis of antioxidants, which demonstrated predictive ability through statistical analysis. However, based on the available data, we could not confirm the predictive capability of the miRNA analysis, and thus we did not include it in the expansion of the diagnostic model.

## Author contributions

**Conceptualization:** Zuzana Drotárová, Mária Mareková, Peter Kizek.

**Data curation:** Zuzana Drotárová, Miroslava Rabajdová, Janka Vašková.

**Formal analysis:** Zuzana Drotárová, Miroslava Rabajdová, Janka Vašková.

**Investigation:** Zuzana Drotárová, Miroslava Rabajdová, Janka Vašková.

**Methodology:** Zuzana Drotárová, Miroslava Rabajdová, Janka Vašková.

**Supervision:** Mária Mareková, Peter Kizek.

**Validation:** Mária Mareková, Peter Kizek.

**Writing – original draft:** Zuzana Drotárová, Janka Vašková.

**Writing – review & editing:** Zuzana Drotárová, Miroslava Rabajdová, Mária Mareková, Peter Kizek, Janka Vašková.
